# Vitamin D status in Norwegian children and associations between child vitamin D status, dietary factors, and maternal vitamin D status

**DOI:** 10.29219/fnr.v69.10727

**Published:** 2025-02-05

**Authors:** Anne Lene Kristiansen, Jannicke Borch Myhre, Linn Kristin Lie Øyri, Kirsten B. Holven, Lene Frost Andersen

**Affiliations:** 1 Department of Nutrition, Institute of Basic Medical Sciences, University of Oslo, Oslo, Norway; 2 Department of Health and Care, Kvam Municipality, Norway; 3 Norwegian National Advisory Unit on Familial Hypercholesterolemia, Department of Endocrinology, Morbid Obesity and Preventive Medicine, Oslo University Hospital, Oslo, Norway

**Keywords:** infant vitamin D status, child vitamin D intake, child dietary sources of vitamin D, Norway, dried blood spot

## Abstract

**Background and aims:**

There is limited data regarding the vitamin D status of infants and young children in Norway. We aimed to assess vitamin D status among Norwegian children at approximately 6 and 12 months of age and explore associations between child vitamin D status, dietary factors, and maternal vitamin D status.

**Methods:**

Mothers/parents completed a food frequency questionnaire for their 6/12-month-old child. Dried blood spot samples were collected from the mother and child.

**Results:**

The mean serum 25-hydroxyvitamin D (S-25(OH)D) concentration was 81 nmol/L (standard deviation [SD] 22 nmol/L) for 6-month-old children (*n* = 84) and 72 nmol/L (SD 22 nmol/L) for 12-month-old children (*n* = 56) (*P* = 0.03 for difference between age groups). In the younger and older age groups, 94 and 88% of the children, respectively, had a S-25(OH)D concentration ≥ 50 nmol/L. The mean dietary vitamin D intake was 12 μg/day for the 6-month-olds and 14 μg/day for the 12-month-olds. Adjusted linear regression models showed that for every μg/day increase in dietary vitamin D intake, serum 25(OH)D (nmol/L) increased by around one nmol/L for both age groups (*P* = 0.002 for the younger age group and *P* = 0.04 for the older age group). Use of vitamin D supplements was associated with higher S-25(OH)D concentrations in both age groups, while a higher S-25(OH)D concentration among formula users was found only in the youngest age group. Breastfeeding was not associated with S-25(OH)D concentration in either age group. Small positive correlations between child and maternal vitamin D status were observed for both the younger (*r* = 0.22) and the older (*r* = 0.28) age groups (*P* = 0.04 for both groups).

**Conclusion:**

While there was a wide range in S-25(OH)D concentrations among children, most were within the sufficient range. Adequate vitamin D intake should be encouraged both in the first and second year of life.

## Popular scientific summary

- Limited information is available concerning the vitamin D status of young Norwegian children. This study measured vitamin D status and intake in 140 children aged around 6 and 12 months.
- Most children had a serum 25-hydroxyvitamin D (S-25(OH)D) concentration higher than 50 nmol/L. The mean S-25(OH)D concentration was lower in the older age group than in the younger age group.
- Child S-25(OH)D concentration was correlated to dietary vitamin D intake and maternal S-25(OH)D concentration.

Vitamin D is a crucial nutrient involved in the regulation of calcium and phosphate metabolism and is therefore essential for the development and maintenance of bone health (1, 2). It is well established that vitamin D deficiency may lead to impaired mineralization of the bone and thereby rickets in children and osteomalacia in adults (3). On the other hand, a high-serum 25-hydroxyvitamin D (S-25(OH)D) concentration may be toxic and lead to hypercalcemia (1).

Over the past decades, several health benefits not directly linked to bone metabolism have been associated with sufficient vitamin D status, such as a reduced risk of cardiovascular disease, type 2 diabetes mellitus, cancer, adverse pregnancy-related outcomes, and autoimmune diseases (4–6). Although results are largely inconclusive, several reviews have suggested a modest protective effect of vitamin D on cancer mortality and/or total mortality (1, 4–6).

Serum 25(OH)D is considered a reliable biomarker of body stores of vitamin D in humans, capturing all sources of vitamin D: diet, dietary supplements, and skin synthesis (1, 3), with a half-life of some weeks (7). There is currently no consensus regarding the optimal S-25(OH)D concentration and cut-off values for defining deficiency, insufficiency, and sufficiency (1, 3). The European Food Safety Authority (EFSA) recently stated that there is evidence for an increased risk of adverse musculoskeletal health outcomes for adults, infants, and children as well as increased risk of adverse pregnancy-related health outcomes for pregnant women at S-25(OH)D concentrations below 50 nmol/L (8). Moreover, the scoping review of vitamin D for the Nordic nutrition recommendations 2023 stated that S-25(OH)D concentrations of less than 25–30 nmol/L are an indication of deficiency (1). With regard to possibly harmful S-25(OH)D concentrations, EFSA has stated that serum concentrations of 200 nmol/L or below are unlikely to pose a risk of adverse health outcomes in infants (9), and that, in general, concentrations higher than ≥ 200–220 nmol/L are necessary to result in vitamin D toxicity (8). In the US, the National Institutes of Health has stated that S-25(OH)D concentrations higher than 125 nmol/L are linked to potential adverse effects, particularly at concentrations higher than 150 nmol/L (10).

There are few natural dietary sources of vitamin D, and they include fatty fish, egg yolk, and some wild mushrooms (1, 11). Additionally, various fortified food products are available depending on country fortification policies. EU legislation applies to the Nordic countries requiring infant formula to contain at least 0.48 μg vitamin D per 100 kJ and cereal based baby foods with an added high protein food (such as baby porridges containing dried milk) to contain at least 0.25 μg vitamin D per 100 kJ (12, 13). Hence, these foods are also potentially important vitamin D sources for the youngest children. Vitamin D can be synthesized in the skin when exposed to ultraviolet B light from the sun (14). However, at higher latitudes, sun exposure is not strong enough to cause vitamin D production during certain months of the year. For example, in Tromsø, Norway (69°N), this period lasts for about 8 months, while it lasts for about 6 months in Oslo, Norway (60°N) (15).

The vitamin D content of breast milk is low (1, 9), and infants are therefore especially prone to vitamin D deficiency (16). Therefore, supplementing infants with vitamin D is recommended in several countries (17), including in Norway (1, 18). For exclusively breastfed infants, a supplement containing 10 μg vitamin D per day is recommended from the age of 1 week. For infants receiving only formula, supplementation is not necessary due to the vitamin D content of the formula. Partly breastfed infants should receive vitamin D supplementation depending on the amount of formula they are fed to meet the total recommended intake of 10 μg/day.

There are limited data available regarding vitamin D status of infants and young children in Norway. A recent mini review of vitamin D status of Norwegian children and adolescents reported vitamin D insufficiency to be prevalent, particularly among non-Western immigrants and adolescents (19). However, little information exists about the vitamin D status of children born to Norwegian mothers. The primary aim of the present study was to assess vitamin D status in non-immigrant Norwegian children around age 6 and 12 months, measured as S-25(OH)D. The secondary aim was to explore the association between child vitamin D status and dietary intake, supplement use, formula use, breastfeeding, and maternal vitamin D status.

## Methods

### Subjects and design

**Fig. 1:**
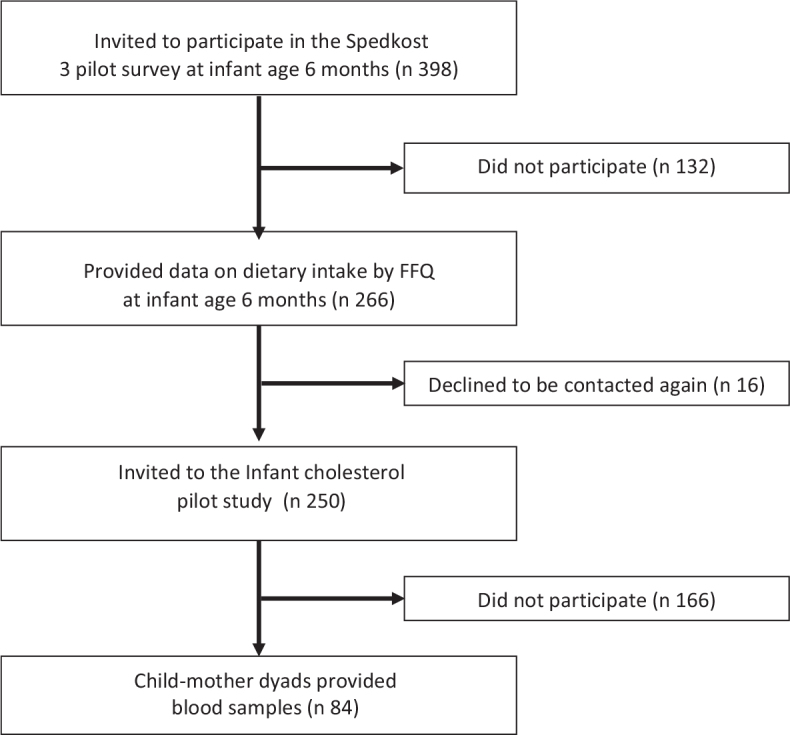
Flow chart of participants in the younger age group.

**Fig. 2:**
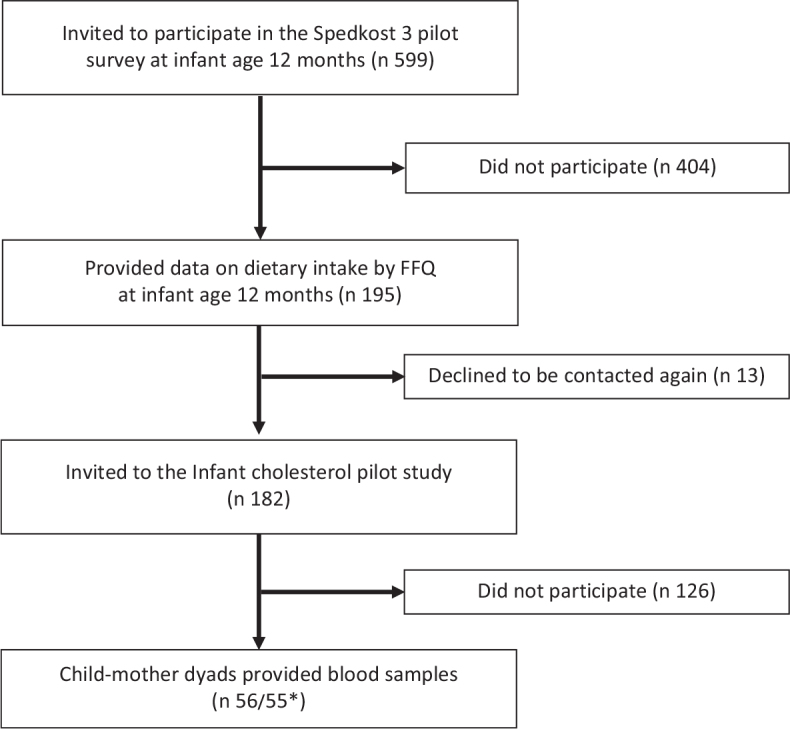
Flow chart of participants in the older age group. *Missing blood value for one mother in the older age group.

Data obtained from secondary analysis presented by Øyri and collaborators (20) were used in the present study. The study design has been described previously (20). In short, the recruitment of subjects was conducted in two steps. At first, two nationwide samples were drawn from the Norwegian National Registry. As presented in the flow chart in Fig. 1, the first sample consisted of 398 children, born during the first days of March 2017. The second sample, presented in the flow chart in Fig. 2, included 599 children born during the first week of September 2016. In the first sample, the mothers/parents received an invitation around child age 6 months, while in the second sample, mothers/parents received an invitation around child age 12 months. Only children born to mothers who themselves were born in Norway, Sweden, or Denmark were included in the drawn samples. Also, a cell phone number had to be registered on the mother for the child/mother dyads to be included.

In the first sample, the mothers/parents were invited to fill in an online semi-quantitative food frequency questionnaire (FFQ) about the child’s diet around age 6 months. The FFQ asked about breastfeeding and complementary feeding, including the use of infant formula, baby porridge, other baby foods, and some foods and beverages. Questions about consumed amounts were included for infant formula, milk, beverages, and supplements. In addition, one question asked about the regular portion of industry-made porridge and homemade porridge. The FFQ did not aim to calculate daily intakes of energy and nutrients due to the importance of breastfeeding to the total dietary intake among this age group.

In the second sample, the mothers/parents were invited to fill in a FFQ about the child’s diet around age 12 months. The FFQ asked about frequencies and amounts for commonly consumed foods and beverages, including supplements, thus intake of energy and nutrients could be calculated in this sample. The FFQ also included questions about current and previous breastfeeding.

For both children aged 6 and 12 months, breastfeeding was dichotomized into breastfed or not.

Both questionnaires were pilot questionnaires (the Spedkost 3 pilot surveys) to be used in the third national dietary survey among children in 2018–19 (the Spedkost 3 surveys) (21, 22). Data collection for the FFQ data was carried out from September to November 2017.

The last question in both FFQs asked if the participant could be contacted again for further studies. Those who agreed to this question were invited to participate in the Infant cholesterol pilot study, which was the second step of recruitment. That study consisted of taking a blood sample by a finger prick from both mother and child by the dried blood spot (DBS) method (described below). Invitations and the DBS kit were sent by mail to the participants. Data collection for this part of the study was carried out from September to December 2017.

### Estimation of dietary intake of vitamin D by the FFQ

For the children aged 6 months, dietary vitamin D was estimated based on the reported intake of vitamin D containing supplements, fortified infant formula, and fortified baby porridge. The questionnaire asked for producer-specific baby porridges, but not for details concerning what kind of porridge within the specific producer the child consumed. Hence, a mean vitamin D value for the existing varieties for the relevant age group from each producer was calculated. As porridges were only quantified using one portion size for all industry-made porridges and one portion size for homemade porridges, these portion sizes were used for all reported porridges. The contribution of vitamin D from other foods, like fish containing baby foods, was not included as only frequency questions (without estimates of portion size) were included in the questionnaire for these foods. Likewise, we did not attempt to estimate vitamin D intake from breast milk. For the children aged 12 months, vitamin D intake was calculated based on the frequencies and amounts reported in the FFQ. As for children aged 6 months, no estimation of vitamin D intake from breast milk was performed for the children aged 12 months.

### Estimation of vitamin D status by the DBS kit

The DBS procedure was performed unsupervised at home. The participants were provided with both written instructions and video instructions on how to perform the procedure. Blood drops from the fingertips of both mother and child were placed on separate filter papers with five pre-marked circles in each paper. The mothers were instructed to let the filter papers dry for 2–4 h after blood application and then put them in a prelabeled aluminum bag containing a small pouch of drying agent. Mothers were requested to return the aluminum bag by mail at the latest the next day. The filter papers were frozen (−80°) within 10 days after sampling. Serum concentrations of vitamin D were quantified using liquid-chromatography-tandem mass spectrometry (LC-MS/MS), which is considered as the gold standard (8). The limit of quantification for the performed analyses was 5 nmol/L. The analysis was done at an accredited medical laboratory, Vitas AS in Oslo (www.vitas.no), and the protocol for the vitamin D analysis was as published by Madar and collaborators (23) and followed general guidelines for bioanalytic methods as described by the U.S. Food and Drug Administration (24). Vitas AS takes part in the Vitamin D External Quality Assessment Scheme (DEQAS).

### Reference values for vitamin D status and intake

Based on the literature (1, 8, 9), S-25(OH)D concentrations < 30 nmol/L were defined as deficiency, concentrations between 30 and 49.9 nmol/L as mild insufficiency, and concentrations ≥ 50 nmol/L as sufficiency. At the high end of the range of S-S-25(OH)D concentrations, the measured concentrations were compared to the suggested ≥ 200 nmol/L that by EFSA was found unlikely to pose a risk of adverse health outcomes as well as to the 125–150 nmol/L suggested by the Institute of Medicine (10). These reference values were applied to both mothers and children.

With regard to vitamin D intake, the estimated vitamin D intake from the FFQs was compared to the recommended intake of 10 μg/day (25). EFSA has published tolerable upper intake levels (ULs) for vitamin D of 25 μg/day for children up to 6 months and 35 μg/day for children aged 6–12 months (8). Hence, vitamin D intakes were also compared to these ULs.

### Ethics

This study was conducted according to the guidelines laid down in the Declaration of Helsinki, and all procedures involving human subjects were approved by the Regional Committees for Medical and Health Research Ethics southeast region of Norway (ref. number 2017/980, the Infant cholesterol pilot study) and the Norwegian Center for Research Data (ref. number 53936, the Spedkost 3 pilot survey). A written-informed consent was obtained from all parents, both for the dietary survey and for the collection of the DBS samples.

### Data analyses

All analyses were conducted using SPSS (IBM Corp.; Released 2022. IBM SPSS Statistics for Windows, Version 29.0. IBM Corp.). Descriptive statistics were presented as means, standard deviation (SD), and range. Pearson correlations (*r*) and scatter plots were used to describe the relationship between child S-25(OH)D concentration and child dietary intake of vitamin D in addition to the relationship between child and maternal S-25(OH)D concentrations. Linear regression analyses were used to explore the relationship between child S-25(OH)D concentration and the following covariates: dietary intake of vitamin D (continuous), use of vitamin D supplement (three categories: not using supplements, using supplements with < 10 μg vitamin D/day, and using supplements with ≥ 10 μg vitamin D/day), formula use (yes vs. no), use of vitamin D containing baby porridge (yes vs. no), total vitamin D intake fulfilling the recommended 10 μg/day or not, and breastfeeding status at 6/12 months of age (yes vs. no). Unadjusted linear regression analyses were conducted to explore the relationships between dietary intake of vitamin D, breastfeeding (yes vs. no), formula use (yes vs. no), and use of vitamin D supplements (three categories as described above). All regression analyses with child S-25(OH)D concentration as dependent variable were adjusted for maternal S-25(OH)D concentration (continuous) and breastfeeding status (yes vs no). Residuals were inspected to verify model assumptions.

## Results

Of the 432 mothers/dyads who were invited to the blood sampling part of the study, 84 children participated in the younger age group (Fig. 1), and 56 children participated in the older age group (Fig. 2). Characteristics of the study samples are shown in Table 1. For children in the younger age group, mean age at dietary reporting was 6.4 months (SD 0.2), while mean age at blood sampling was 7.7 months (SD 0.9). The corresponding values for children in the older age group were 12.4 months at dietary reporting (SD 0.3) and 13.8 months at blood sampling (SD 0.8). The minimum and maximum number of days between completing the FFQ and the blood sampling were 6 and 90 days, respectively (data not shown). Participants were mostly from the eastern and western parts of Norway (~60ºN). Mean maternal age was 31 years in the younger age group and 32 years in the older age group.

**Table 1:** Background characteristics of children and mothers

Background variable	Children in the younger age group (*n* = 84)	Children in the older age group (*n* = 56)
**Child gender**		
Boys (*n*, %)	41 (49)	38 (68)
Girls (*n*, %)	43 (51)	18 (32)
**Child anthropometrics at dietary reporting**		
Weight (kg) (mean, SD)	8.1 (1.2)	10.2 (1.2)
Height (cm) (mean, SD)	68 (4)	77 (3)
**Child age at dietary reporting**		
Months (mean, SD)	6.4 (0.2)	12.4 (0.3)
**Child age at blood sampling**		
Months (mean, SD)	7.7 (0.9)	13.8 (0.8)
**Region of the country and latitude**		
East (~60ºN) (*n*, %)	36 (43)	25 (45)
West (~60ºN) (*n*, %)	25 (30)	19 (34)
South (~58ºN) (*n*, %)	4 (5)	3 (5)
Middle (~63ºN) (*n*, %)	11 (13)	2 (4)
North (~69ºN) (*n*, %)	8 (10)	7 (13)
**Maternal age**		
Years (mean, SD)	31 (4)	32 (6)

### Vitamin D status among children and mothers

The mean S-25(OH)D concentration among children in the younger age group was 81 nmol/L (SD 22), ranging from 32 to 149 nmol/L (Table 2). A significantly lower S-25(OH)D concentration (*P* = 0.03) was seen among children in the older age group, with a mean serum concentration of 72 nmol/L (SD 22), ranging from 12 to 165 nmol/L (Table 2). None of the children in the younger age group, but one child in the older age group was classified as having vitamin D deficiency (S-25(OH)D < 30 nmol/L). Eleven children were considered to have mild vitamin D insufficiency (S-25(OH)D between 30 and 49.9 nmol/L), five children in the younger age group, and six children in the older age group (Table 3). Four children (two in the younger and two in the older age groups) had an S-25(OH)D concentration above 125 nmol/L, and one child had an S-25(OH)D concentration above 150 nmol/L. None of the children had an S-25(OH)D concentration higher than 200 nmol/L (Table 3).

**Table 2:** Serum 25(OH)D concentration (nmol/L) among children and mothers^1^ in relation to selected characteristics

Characteristic	Children in the younger age group (*n* = 84)				Children in the older age group (*n* = 56)			
	*n*	Serum 25(OH)D concentration, nmol/L, mean (SD)	Range	*P* ^1^	*n*	Serum 25(OH)D concentration, nmol/L, mean (SD)	Range	*P* ^1^
**Serum concentration**								
Child	84	81 (22)	32–149		56	72 (22)	12–165	
Mother^2^	84	69 (22)	30–129		55	64 (25)	28–144	
**Child region of the country and latitude** ^3^								
East (~60ºN)	36	82 (20)	37–124		25	80 (25)	43–165	
West (~60ºN)	25	80 (23)	36–149		19	66 (13)	45–87	
South (~58ºN)	4	75 (19)	65–103		3	70 (10)	62–81	
Middle (~63ºN)	11	75 (26)	32–126		2	86 (-)^4^	-^4^	
North (~69ºN)	8	90 (29)	46–117		7	55 (23)	12–80	
**Maternal region of the country and latitude** ^3^								
East (~60ºN)	36	70 (22)	33–129		25	66 (25)	31–122	
West (~60ºN)	25	75 (23)	35–119		19	64 (24)	28–130	
South (~58ºN)	4	59 (5)	53–64		3	49 (10)	42–61	
Middle (~63ºN)	11	66 (23)	30–100		2	103 (-)^4^	-^4^	
North (~69ºN)	8	52 (7)	41–63		7	55 (16)	36–79	
**Breastfeeding status**				0.37				0.96
Breastfed	70	80 (23)	32–149		26	71 (28)	12–165	
Not breastfed	14	84 (17)	51–117		30	73 (15)	45–108	
**Formula use**				0.03				0.92
Yes	21	89 (18)	51–123		21	73 (17)	43–108	
No	63	78 (23)	32–149		35	71 (25)	12–165	
**Vitamin D containing baby porridge use** ^5^				0.43				1.00
Yes	61	83 (22)	32–149			72 (23)	12–165	
No	23	76 (24)	36–124			71 (14)	45–86	
**Total vitamin D intake** ^6^				0.16				0.03
< 10 μg/day	28	75 (23)	36–149		15	62 (20)	12–87	
≥ 10 μg/day	56	84 (22)	32–126		41	75 (22)	43–165	
**Use of vitamin D supplement**				0.03				0.07
No use of supplement	12	69 (20)	36–116		14	65 (22)	12–87	
< 10 μg/day	31	77 (21)	32–149		17	66 (9)	48–82	
≥ 10 μg per day	41	88 (22)	46–126		25	80 (26)	46–165	

1 Linear regression adjusted for maternal serum 25(OH)D concentration (nmol/L) and breastfeeding status (yes vs. no).

2 Missing blood value for one mother in the older age group.

3 Differences between regions not tested statistically due to a low number of participants living in some of the regions.

4 SD and range not calculated due to small sample size.

5 To be included as a user of vitamin D-containing baby porridge in the intake of vitamin D from porridge was at least 0.5 μg/day,

6 Total dietary vitamin D intake includes vitamin D from dietary supplements, formula, baby porridge, and other foods (only the children aged 12 months have data for ‘other foods’).

**Table 3:** Serum 25(OH)D concentration (nmol/L) among children and mothers^1^ in relation to reference values

Serum 25(OH)D group	Serum 25(OH)D cut-offs (nmol/L)	Children in the younger age group (*n* = 84) *n* (%)	Children in the older age group (*n* = 56)^1^ *n* (%)
**Deficiency**	< 30		
Child		0	1 (2)
Mother		1 (1)	1 (2)
**Mild insufficiency**	30–49.9		
Child		5 (6)	6 (11)
Mother		14 (17)	18 (33)
**Sufficiency**	≥ 50		
Child		79 (94)	49 (88)
Mother		69 (82)	36 (65)
**Potentially high concentration**			
**Higher than 125 nmol/L**	125		
Child		2 (2)	2 (4)
Mother		1 (1)	2 (4)
**Higher than 150 nmol/L**	150		
Child		0	1 (2)
Mother		0	0
**Higher than 200 nmol/L**	200		
Child		0	0
Mother		0	0

1 Missing blood value for one mother in the older age group.

The S-25(OH)D concentration did not differ according to breastfeeding status in neither of the groups (Table 2). In the younger age group, a higher serum concentration was seen in formula users compared with children not using formula (mean difference 11 nmol/L, *P* = 0.03). This difference was not seen in the older age group (Table 2). In both age groups, those with a total dietary intake of vitamin D of below 10 μg/day had a lower mean S-25(OH)D concentration compared to those with a dietary intake of vitamin D of ≥ 10 μg/day (mean difference 10 nmol/L, *P* = 0.16 for the 6 month olds and mean difference 13, *P* = 0.03 for the 12 month olds). Also, in both age groups, those with the highest reported intake of vitamin D from supplements had the highest mean S-25(OH)D concentration (the mean difference between non-users of supplements and those taking supplements with ≥10 μg/vitamin D/day was 19 nmol/L (*p* = 0.03) for the 6 month olds and 16 nmol/L (*p* = 0.04) for the 12 month olds, Table 2).

The mean maternal S-25(OH)D concentration was 69 (SD 22) nmol/L, with a range of 30–129 nmol/L among mothers of children in the younger age group, whereas the mean concentration was 64 (SD 25) nmol/L, ranging from 28 to 144 nmol/L among mothers of children in the older age group (Table 2). Two mothers were classified as having vitamin D deficiency, one in each age group (Table 3), while 14 mothers in the younger age group and 18 mothers in the older age group were classified as having mild vitamin D insufficiency (Table 3). Moreover, three mothers had S-25(OH)D concentrations higher than 125 nmol/L, but none of the mothers had concentrations higher than 150 nmol/L.

**Fig. 3:**
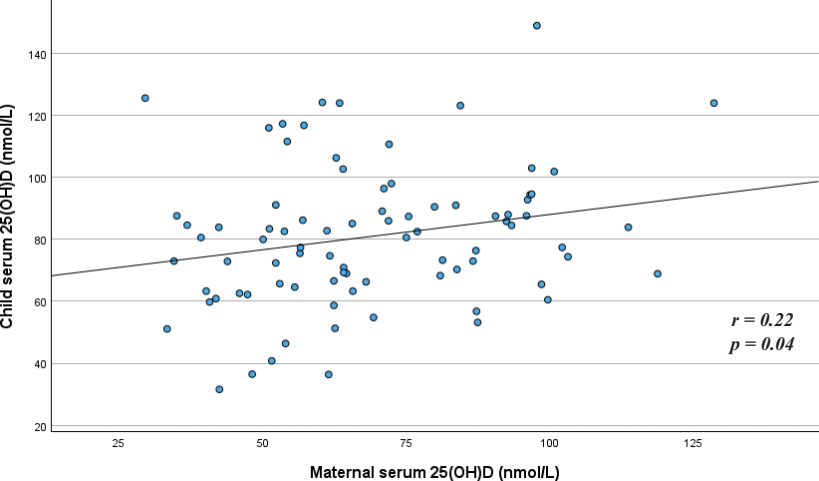
Relationship between child and maternal serum 25(OH)D concentration among children in the younger age group (*n* = 84). ^*^Pearson correlation.

**Fig. 4:**
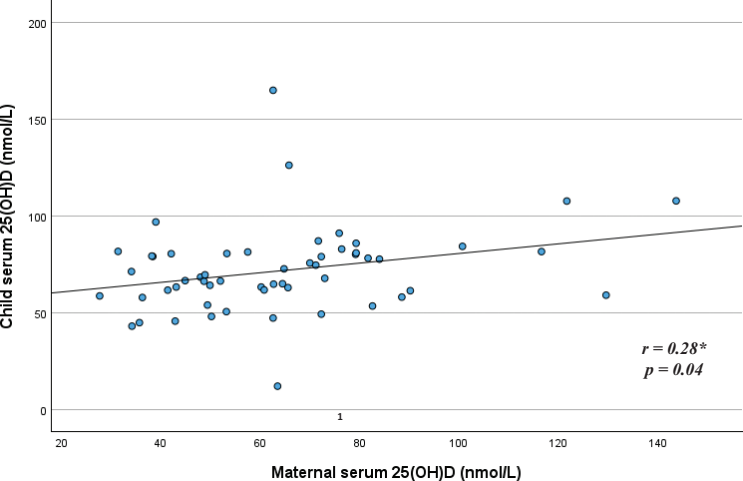
Relationship between child and maternal serum 25(OH)D concentration among children in the older age group (*n* = 55**). *Pearson correlation. **Missing blood value for one mother in the older age group.

When exploring the relationship between child and maternal S-25(OH)D concentrations, small positive correlations were observed for both the younger (*r* = 0.22, *P* = 0.04) (Fig. 3) and the older (*r* = 0.28, *P* = 0.04) age groups (Fig. 4).

### Dietary intake of vitamin D

Children’s dietary intakes of vitamin D, estimated from the FFQ, are presented in Table 4. The mean vitamin D intake among children in the younger age group was 11.6 μg/day (SD 7.0), ranging from 0.1 to 35.0 μg/day. Four children had total vitamin D intakes exceeding the tolerable UL of 25 μg vitamin D/day for children up to 6 months. Among children in the older age group, the mean intake was 13.9 μg/day (SD 6.7), with a range from 1.1 to 32.1 μg/day. None of the children had total vitamin D intakes exceeding the UL of 35 μg vitamin D/day for children between 6 and 12 months.

**Table 4:** Dietary intake of vitamin D (μg/day) estimated by the FFQ in relation to selected characteristics

	Children in the younger age group (*n* = 84)				Children in the older age group (*n* = 56)			
	*n*	Vitamin D, μg/day, mean (SD)	Range	*P* ^1^	*n*	Vitamin D, μg/day, mean (SD)	Range	*P* ^1^
**Total vitamin D intake** ^2^				NA				NA
μg/day (Mean, SD)	84	11.6 (7.0)	0.1–35.0		56	13.9 (6.7)	1.2–32.1	
**Breastfeeding**				< 0.001				0.05
Breastfed	70	10.3 (6.1)	0.1–30.8		26	12.0 (6.7)	2.8–30.1	
Not breastfed	14	18.5 (7.3)	9.3–35.0		30	15.5 (6.5)	1.2–32.1	
**Formula user**				< 0.001				0.02
No	63	9.7 (5.7)	0.1–30.0		35	12.2 (6.8)	1.2–30.1	
Yes	21	17.4 (7.4)	8.6–35.0		21	16.7 (5.8)	7.9–32.1	
**Vitamin D supplement use**				< 0.001				< 0.001
No use of supplement	12	5.6 (5.8)	0.1–15.1		14	7.7 (5.3)	1.2–19.7	
< 10 μg/day	31	9.0 (4.4)	2.9–18.4		17	11.5 (3.7)	5.4–18.1	
≥ 10 μg per day	41	15.4 (6.9)	10.0–35.0		25	19.0 (5.1)	11.9–32.1	
**Vitamin D sources**	84			NA	56			NA
Dietary supplements		7.9 (5.4)	0–28.3			6.6 (5.3)	0–20.0	
Baby porridge		2.0 (2.1)	0–7.9			3.2 (2.4)	0–10.6	
Formula		1.8 (3.9)	0–21.6			1.6 (2.9)	0–15.4	
Other foods		NA	NA			2.6 (1.8)	0.2–9.7	

1 Linear regression.

2 Total dietary vitamin D intake includes vitamin D from dietary supplements, formula, baby porridge, and other foods (only the children aged 12 months have data for ‘other foods’).

The mean dietary intake of vitamin D in relation to selected characteristics was comparable in the two age groups (Table 4), and results are therefore presented together in the following section. Non-breastfed children had higher vitamin D intake than those who were breastfed. Correspondingly, users of infant formula had a higher intake of vitamin D compared to non-users. Additionally, those with the highest reported intake of vitamin D from supplements (≥ 10 μg per day) had the highest total intake of vitamin D (Table 4).

### Important dietary sources of vitamin D

In both age groups, dietary supplements were by far the largest single source of dietary vitamin D, contributing with 7.9 μg/day (68%) of vitamin D in the younger age group and 6.6 μg/day (47%) in the older age group (Table 4). Baby porridge was also an in important source of vitamin D, contributing with 2.0 μg/day (17%) in the younger age group and 3.2 μg/day (23%) in the older age group. Formula contributed with 1.8 μg/day (15%) of dietary vitamin D in the younger age group and 1.6 μg/day (11%) in the older age group. Among children in the older age group, other foods contributed with 2.6 μg/day (19%) of dietary vitamin D (Table 4).

### Correlations between vitamin D intake and vitamin D status

**Fig. 5:**
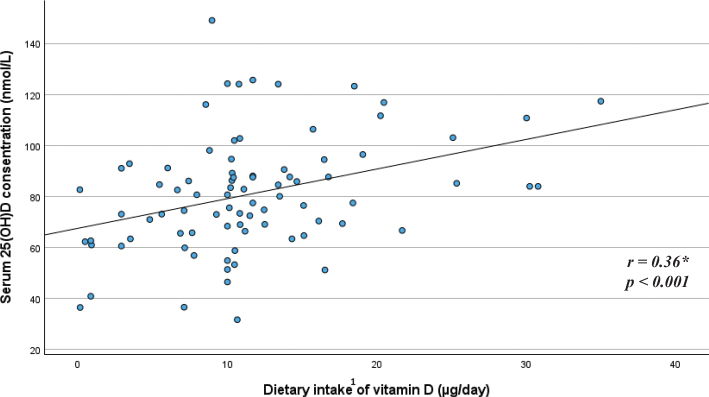
Relationship between child serum 25(OH)D concentration and child dietary intake of vitamin D among children in the younger age group (*n* = 84). ^*^Pearson correlation. ^1^Total dietary vitamin D intake includes vitamin D from dietary supplements, formula, baby porridge, and other foods.

**Fig. 6:**
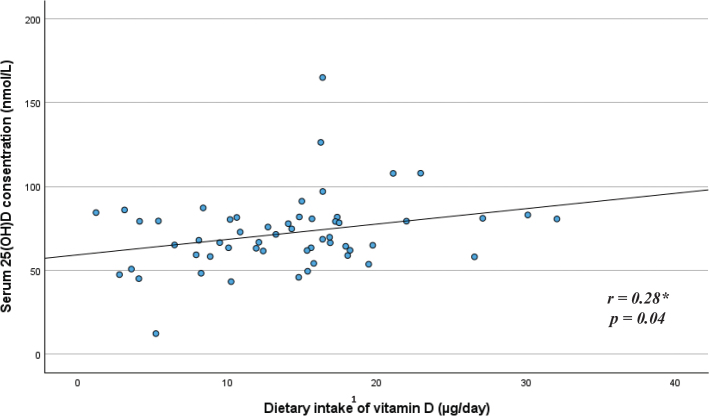
Relationship between child serum 25(OH)D concentration and child dietary intake of vitamin D among children in the older age group (n 56), around child age 12 months. *Pearson correlation. ^1^Total dietary vitamin D intake includes vitamin D from dietary supplements, formula, baby porridge, and other foods.

Exploring the relationship between child S-25(OH)D concentration and the child dietary intake of vitamin D, a medium positive correlation was observed for the younger age group (*r* = 0.36, *P* < 0.001) (Fig. 5), and a small positive correlation was observed for the older age group (*r* = 0.28, *P* = 0.04) (Fig. 6). Adjusted linear regression models showed that for every μg/day increase in dietary intake of vitamin D, S-25(OH)D (nmol/L) increased by around one nmol/L for both age groups (*P* < 0.002 for the younger age group and *P* = 0.04 for the older age group) (data not shown).

## Discussion

The results from the present study showed that there was a wide range in S-25(OH)D concentrations among children; however, most were within the sufficient range. In both age groups, vitamin D intake from the diet was positively associated with vitamin D status. Dietary supplements were the single largest contributor to dietary vitamin D intake. For both age groups, a small positive correlation between child and maternal vitamin D status was found.

When applying a sufficiency level of ≥ 50 nmol/L to the measured S-25(OH)D concentrations in the present study, the proportion of children in the sufficient range was somewhat higher in the younger age group (94%) compared with the older age group (88%). The mean S-25(OH)D concentration was also higher among children in the younger age group (81 nmol/L) compared with children in the older age group (72 nmol/L). There are some available data regarding vitamin D status among children in this age group from the Nordic countries. A study including 255 Danish infants at 9 months of age reported a mean serum concentration of 77 nmol/L with 89% of the infants having S-25(OH)D concentrations above 50 nmol/L (26). A study from Iceland among 76 infants at 12 months of age reported a mean S-25(OH)D concentration of 98 nmol/L with 92% of the children having serum concentrations above 50 nmol/L (27). In Norway, in 2000, a study among 1-year-old children in Oslo showed 20–34% of the children having S-25(OH)D concentrations under 50 nmol/L (28), while a somewhat more recent study from Western Norway among 62 1-year olds in 2011/12 showed a mean S-25(OH)D concentration of 85 nmol/L with all children having a vitamin D concentration of more than 50 nmol/L (29). In a slightly older group of children, a study from 2001 among 225 2-year-olds in Oslo found a mean serum concentration of 66 nmol/L and 23% of the children having S-25(OH)D concentrations below 50 nmol/L (30). Moreover, a Norwegian register-based study covering the years from 2008 to 2012 identified very few cases of rickets from medical records, indicating a low prevalence of severe vitamin D deficiency in Norwegian children (31). Our data therefore seem to be in line with previous data; however, comparability across studies may be limited as variations in the results of S-25(OH)D measurements across different laboratories have been observed (11).

There was a wide range in S-25(OH)D concentrations among children in the present study, and this has also been reported in previous studies (26–30). We did not have information about exposure to sunlight, use of sunscreen, etc., which are important factors that can impact S-25(OH)D concentrations (15). The study among Danish infants at 9 months of age reported seasonal differences in serum vitamin D concentrations (26), while the study among Islandic infants at 12 months did not find this (27). In Norway, it is recommended to keep infants below the age of 12 months out of direct sunlight, while lightweight clothing and avoidance of long periods of exposure of direct sunlight are recommended for children between 1 and 3 years of age (32). Moreover, the DBS samples were collected from September to December when the intensity of UV radiation is low. We therefore assume that sun exposure most probably contributed minimally to the vitamin D concentrations in the studied group of children (15). Hence, the relatively high mean serum concentrations could mostly be explained by a large proportion of the children regularly using vitamin D supplements and/or fortified food products.

Both correlation analysis and adjusted linear regression models showed that there was a positive association between child S-25(OH)D concentration and child dietary intake of vitamin D in both age groups. Dietary supplements were by far the largest single source of dietary vitamin D in both age groups, used by 86% in the younger age group and 75% in the older age group. The study among 12-month-old Icelandic infants also reported a difference in vitamin D status between users and non-users of vitamin D supplements (27). Additionally, the study among Norwegian 2-year-olds found that users of vitamin D supplements had a higher mean S-25(OH)D concentration (75 nmol/L) compared to non-users (60 nmol/L) (30). Such results were not found among 9-month-old Danish infants (26) or among the 1-year-olds from the western part of Norway (29). However, in those studies, 97 and 80% of the children used vitamin D supplements.

The mean S-25(OH)D concentration was about 10 nmol/L higher among children in the younger age group compared with children in the older age group. This might partly be caused by a lower proportion of supplement users among the older children. The percentage of vitamin D supplement users among the older age group in the present study was in line with what has been reported in the Norwegian national dietary survey among infants at 12 months of age (72%) (33). However, the most recent national dietary survey among Norwegian 2-year-olds showed that only 54% of the children used vitamin D supplements, suggesting a lower percentage of users with increasing age (34). There might be more focus on vitamin D supplementation during the first year of life compared to later in childhood. Around 1 year of age, children are likely to replace the fortified infant formula with cow’s milk. In Norway, some semi-skimmed milks are lightly fortified with vitamin D, while others are not. This might lead to a lower intake of vitamin D unless vitamin D supplementation is increased. If supplement use in this age group also is reduced, as indicated in the present study, the proportion of children with suboptimal vitamin D intakes may increase. Hence, based on our results, it seems important to promote the use of vitamin D supplements, particularly for children around 1 year of age.

In infancy, infant formula is often an important contributor of dietary vitamin D (26, 27), and this was seen in both age groups in the present study. Adjusted analysis among children in the younger age group showed that children who received formula had higher serum concentration of vitamin D compared with children who did not receive formula. Such associations were also observed among Danish infants at 9 months of age (26).

We did not find any associations between breastfeeding and vitamin D status in either age group. Similar observations have been reported in two studies among children at 12 months of age (27, 28). However, breastfeeding at 9 months of age was linked to lower S-25(OH)D concentrations among Danish infants (26). Infants in Norway have, until 2020, been recommended supplementation of vitamin D from the age of 4 weeks, regardless of whether the infant was breastfed or formula fed (18), and this was the recommendation at the time of this study. In 2020, a revised recommendation was launched, and the recommendation for vitamin D supplementation now differentiates according to breastfeeding status (25).

Small positive correlations between child and maternal vitamin D status were observed for both age groups in the present study. Such results were also observed among 1-year-olds from the western part of Norway (29). Correlation in that study was, however, somewhat higher than what was observed in the present study (*r* = 0.4, *P* < 0.05) (29). We do not have information about the diet of the mother in the present study, but some of the association between the child and the maternal vitamin D status might be caused by some shared dietary habits between mother and child.

Eighteen percent of the mothers in the younger age group and 34% of mothers in the older age group had serum values < 50 nmol/L. For the older age group, this was in line with mothers of 1-year-olds from the western part of Norway, where 32% of the mothers had serum values < 50 nmol/L. A nationwide Norwegian study among 2-year-old children and their mothers did only assess vitamin D status in the mothers, and it found that 18% had serum values < 50 nmol/L (35). Itkonen et al. reported that 7–24% of the adult population in the Nordic countries had serum values < 50 nmol/L (11). However, it might be that the mother population have lower vitamin D status than the general population. Possible differences in analytical assays must also be taken into consideration when comparing results from different studies and countries.

### Strengths and limitations

An important strength of the present study was the assessment of both vitamin D status and dietary intake of vitamin D in an understudied age group. Due to the limited data related to vitamin D status among non-immigrant Nordic infants and young children living at a high latitude, the present study contributes to fill this gap. Another strength is the invitation of a nationally representative sample to this study. Even though the dietary data were collected as part of a pilot study, the dietary questionnaires used in the present study were almost identical to the questionnaires used in the national dietary survey among children at 6 and 12 months of age (33, 36). The major circulating form of vitamin D S-25(OH)D, which is considered to be a reliable biomarker of body stores of vitamin D, was assessed. Serum concentration of S-25(OH)D was quantified by the DBS technique, which is a less invasive procedure compared to standard blood-sampling practices. The DBS method also enabled parents to collect blood samples unsupervised at home, making it more feasible to participate. Finally, DBS has been shown to provide a good reflection of vitamin D status, also among infants (37–42). Our results do come with some limitations. The data are cross-sectional, and no causal relationship can be established. Even though the invited participants represented nationwide samples, only about 10 to 20% of those invited decided to participate in both the dietary survey and in the provision of blood samples. Low response rates may therefore have biased our results and led to non-generalizable results. Furthermore, this study included children born to mothers who themselves were born in Norway, Sweden, or Denmark. Therefore, findings do not directly apply to at-risk children of immigrant background. Moreover, the dietary data were self-reported and reliant on the ability of the parent to correctly assess the diet of their child. A previous version of the FFQ used to estimate dietary intake in the 12-month-olds has been found to overestimate the intake of energy and nutrients (43). It is therefore likely that the intake of vitamin D was overestimated in the 12-month-olds in the present study. Due to the recruitment protocol for the blood sampling part of the study, the DBSs were collected almost one and a half months after answering the FFQ. Hence, dietary intake might have changed between the two time points, possibly attenuating the associations between dietary intake and vitamin D status. Another limitation of this study is the lack of information on sun exposure. However, the implication of this is uncertain as it is recommended to keep infants and young children out of direct sunlight.

## Conclusion

In conclusion, there was a wide range in S-25(OH)D concentrations among children; however, most were within the sufficient range, and only a few had vitamin D deficiency or mild insufficiency. Vitamin D intake from the diet was positively associated with vitamin D status. Dietary supplements were the single largest contributor to dietary vitamin D intake, followed by baby porridge and formula. Based on our results, it seems important to promote the use of vitamin D supplements, especially for children around 1 year of age.
